# Safe Travel Practices and Awareness among Diabetic Patients

**DOI:** 10.1155/2023/6353086

**Published:** 2023-01-31

**Authors:** Afnan S. Younis, Noura A. Abouammoh, Layla M. AlBreacan, Yara Aldigi, Atikah Kadi, Nora Almohideb, Nouf AlAmari, Turky H. Almigbal

**Affiliations:** ^1^Department of Family and Community Medicine, College of Medicine, King Saud University, P.O. Box 2925, Riyadh 11461, Saudi Arabia; ^2^College of Medicine, King Saud University, Riyadh 11461, Saudi Arabia

## Abstract

**Objective:**

To measure the knowledge of Saudi patients with diabetes in coping with their condition and to assess their practice of disease control during travels. *Study Design.* Cross-sectional study using a self-administered questionnaire.

**Method:**

This study was conducted between September 2018 and May 2019 at a University hospital in Riyadh, Saudi Arabia. The questions were adopted from guidelines and advices provided by the CDC, American Diabetic Association, and other references. Bivariate and multivariate analyses were used to identify factors associated with diabetic control during travels.

**Results:**

From the included 242 patients, 33.6% showed the good practice of diabetic control during travels. 23.7% of patients were communicated by their doctors about the importance of consultations before traveling and 20.7% encountered complications during travels. Factors associated with doctors' consultation before travel are patients' concerns about travel duration and possible risks during trip. (OR = 2.588, 95% CI = 5.308–1.261), (OR = 3.525, 95% CI = 8.152–1.525); respectively.

**Conclusion:**

Patient awareness and education about the importance of proper diabetic self-monitoring and control during travels is crucial as the study showed suboptimal diabetes management practice. *Practice Implications.* Physicians should proactively educate patients about the importance of seeking advice before their travels.

## 1. Introduction

Modern technology has transformed travel from a need-based activity to a luxurious experience accessible to almost everyone, for business, medical, or personal purposes. In 2015, the tourism movement exceeded all expectations with 1.2 billion international tourist arrivals; this number is expected to rise to 2 billion by 2030 [1]. Meanwhile, the number of people in Saudi Arabia who traveled in 2011 was more than 4 million, increasing to approximately 6 million in 2015; this number is expected to rise further in the future [2].

Diabetes mellitus (DM) is a global health issue responsible for approximately 1.6 million deaths annually; [3] its prevalence has increased dramatically over the years [4];in Saudi Arabia, it is 24%, with an estimated 7 million patients [5, 6]. Furthermore, the growth pattern of this condition is alarming [7].

There is no evidence that diabetic patients are more likely to develop related complications such as vascular or renal disease while traveling than those who do not have DM [3]. However, a change in multiple factors such as diet, level of activity, and environment (temperature and altitude) while traveling can alter blood glucose levels, making it challenging to maintain them [8].

The Centers for Disease Control and Prevention (CDC) provides general and specific advice for diabetic patients looking to travel or traveling, including consulting a healthcare provider for insulin dose adjustment according to their destination, activities, and diet changes, as well as carrying a glucose monitor and insulin at all times [9, 10].

Diabetic patients have the right to travel safely. Therefore, we conducted this study to assess the knowledge and practices of Saudi diabetic patients in terms of managing and coping with their condition while traveling. The results of this study could be beneficial for healthcare services aimed at such travelers, for providing better care and preventing complications.

## 2. Methods

This was a cross-sectional study, conducted at King Khalid University Hospital (KKUH), Riyadh, Saudi Arabia. Data were collected between September 2018 and May 2019 from Saudi diabetic patients aged above 18 and visiting KKUH's diabetic outpatient clinics who had traveled in the last 12 months. Patients with gestational diabetes were excluded.

The sample size was calculated using the standard formula for cross-sectional studies, considering a significance level of 5%, a precession of 7%, and a prevalence of good DM management while traveling of 38% [10]. While the initial sample size required for this study was 185, after adding 10% for the nonresponse rate, it was 205.

Saudi diabetic patients visiting KKUH were recruited from 3 clinics, specialized diabetes clinics that serve the general population in 2 different locations, the family medicine department and internal medicine department, in addition to primary care clinics that serve the university faculty and their dependents.

Using a simple random sampling method, participants were approached from each of the mentioned clinics from different sessions and days of the week in order to give patients an equal chance to be included.

Patients were approached in clinic's waiting area by explaining the need for diabetic patients who had traveled in the last 12 months as study participants; it was also clarified to them that their decision to participate would not affect their consultation with the physician.

Data were collected through a self-administered questionnaire containing questions on demographics including age, gender, education level, and occupation; diabetes and other comorbidities; and travel history, knowledge, and practices. The questions were adopted from the guidelines and advice provided by the CDC and American Diabetic Association, and a survey by Elkins et al. [9, 11, 12] Face and content validity of the questionnaire were assessed by two independent researchers (an epidemiologist and a diabetes consultant).

The study was piloted on 20 patients with diabetes from the general population to test for clarity and applicability of questionnaire; the time needed to fill the questionnaire. Data obtained from the pilot study were not included in this study.

Data were analyzed using SPSS 24.0 version statistical software. Descriptive statistics (mean, standard deviation, frequencies, and percentages) were used to describe the quantitative and categorical variables. The bivariate statistical analysis was carried out using appropriate (chi-square, student's *t*-test) statistical tests. A *p*-value of <0.05 and 95% confidence interval were used to report the results' statistical significance and precision.

A multivariate logistic regression was performed to adjust for confounding variables. Variables with a *p*-value of 0.2 or less in bivariate analysis were entered into the regression model using enter method.

The main outcome was pretravel medical consultation as an indicator of good travel practices. This indicator was further analyzed to determine its effect on the other elements of DM management while traveling, including carrying identification (ID), snacks, medications, and a first aid kit; destination choice, mode of transport; telling one's partner about the condition; self-monitoring; self-control; management issues; and complications.

The consent form was included with each questionnaire copy distributed; it explained the study purpose and included an agreement to participate and the participant's right to withdraw at any time without any obligation.

Participants' autonomy was ensured as well as data confidentiality and anonymity by number coding them for the purpose of analysis.

## 3. Results

The study sample comprised 242 diabetic patients, a majority of whom (84.7%) had type 2, while 56.6% were noninsulin users (Table 1).

Only 23.7% of the respondents' physicians had informed them about the importance of pretravel medical consultation, while 14.9% of the respondents had concerns related to traveling; of these, 82.9% said that consultation with their physician would have helped address their concerns (Table 1).

Regarding overseas travel, 55.5% believed that they could go to a general practitioner for receiving medical care, while 59.7% thought that they would be able to buy their medications at a nearby pharmacy (Table 2).

Assessment of their practices revealed that 33.6% of the respondents had consulted their physician before traveling. Of the respondents who had traveled abroad, 52.5% had exhibited poor self-control. Furthermore, 20.7% had experienced complications while traveling, with 54% of them experiencing hypoglycemia.

A minority of the respondents (7.9%) had carried an item identifying their condition, such as a card, bracelet, or smartphone health app; 67.6% had carried snacks; an overwhelming number (95%) had carried their medications.

Regarding DM's impact on travel, the condition affected destination choice and mode of transport of 12.8% and 18.2% of the respondents respectively (Table 2).

Bivariate analysis showed that the mean age of the respondents who sought medical advice was 48.96 and of those who did not was 53.28, with a statistically significant difference (*p*=0.009, 95% CI = 7.525–1.103).

When comparing the respondents' education, the results revealed that those educated tended to visit their physician before traveling more than their uneducated counterparts (OR = 2.190, 95% CI = 3.814–1.258, *p*=0.005). The longer the trip duration, the higher the likelihood of the respondents visiting their physician (OR = 2.253, 95% CI = 4.010–1.266, *p*=0.005). Likewise, the respondents' concern about possible risks during the trip influenced their decision to visit their physician before traveling (Table 3).

The study also showed that the respondents who consulted their physician were more likely to inform their partner about their condition (OR = 2.850, 95% CI = 7.782–1.004, *p*=0.034), as well as carry ID and a first aid kit (Table 3).

In multivariate analysis, the logistic regression model showed 4 factors significantly associated with DM management while traveling: carrying ID and a first aid kit, and concerns about trip duration and possible risks during the trip. The respondents carrying ID and a first aid kit were more likely to have consulted their physician before traveling (OR = 9.214, 95% CI = 39.732–2.137 and OR = 2.048, 95% CI = 3.867–1.085). Furthermore, those who had concerns about the trip duration and possible risks had visited their physician more often (OR = 2.588, 95% CI = 5.308–1.261 and OR = 3.525, 95% CI = 8.152–1.525) (Table 4).

## 4. Discussion

This study was conducted to investigate the compliance of Saudi diabetic patients with safe travel guidelines and to investigate the factors affecting their behaviors. For this, pretravel medical consultation was used as an indicator of good compliance, observed in only one-third (33.6%) of the respondents.

Factors affecting pretravel medical consultation were concerns about trip duration and potential risks and carrying ID and a first aid kit. This study showed that the respondents who were concerned about trip duration or possible risks demonstrated a higher compliance with safety guidelines and a higher tendency to consult their physician before traveling; this is similar to a finding of the study by Burnett who found that travelers tend to consult their physician before departure when their trip duration is long [13].

Carrying an ID and a first aid kit are important recommendations by healthcare professionals to diabetic patients looking to travel or traveling [9]. Although in this study, the proportions of respondents carrying ID and a first aid kit were low (7.9% and 42.3%), pretravel medical consultation had a significant impact on these behaviors. This showed that those who visited their physician before traveling were more knowledgeable and engaged in significantly better DM management practices as per the recommendations for safe travel.

This study revealed that the respondents' main reason for consulting a physician was to get advice on glucose monitoring during their trip. This result is similar to that of a previous study, which found glucose monitoring to be one of the main reasons the participants went for pretravel medical consultation, in addition to insulin dose adjustment as a majority of that study's participants were insulin users [12]. However, in the present study, the respondents did not know how to adjust their insulin dose, demonstrated by the low response rate to a question on insulin dose adjustment during time zone changes; this finding has been previously observed in different populations, highlighting a general lack of knowledge of the practice of insulin dose adjustment [12, 13].

More than half of the present study's respondents who had experienced complications during their trip had had at least one episode of hypoglycemia. Previous research has attributed this to delayed meal time or infectious diseases [13–15].

In the present study, the majority of the respondents said that they would visit a general practitioner for receiving medical care or go to a nearby pharmacy to buy their medications when abroad. However, American diabetic patients get their medications through the emergency department and ask the hotel staff about where they can go for receiving medical care [12].

Saudi Arabia has a high DM prevalence, which is expected to increase further in the coming years [6]. Among this growing population of patients, a large number may travel multiple times [2]. This study assessed the possible risks encountered by diabetic patients who had traveled and highlighted several areas of importance. It also provides an overview of the behaviors of Saudi diabetic patients who travel and the complications they might face.

This was a hospital-based study, and thus, the results cannot be generalized to the entire population of diabetic patients in Saudi Arabia; therefore, a community-based study including multiple centers from different cities would provide a wider range of responses that could be generalized. There was no validated scoring system measuring knowledge and practices; therefore, items included in assessing knowledge and practice were analyzed individually rather than as a group factor. Both type 1 and type 2 DM were included in this study; however, a community-based study focusing on insulin-using diabetic patients is recommended to investigate their specific needs as they are more prone to risks while traveling.

## 5. Conclusion

In conclusion, the majority of the respondents in this study had not sought out medical advice before traveling; and it was also found that pretravel medical consultation was a good indicator of their overall practices.

### 5.1. Practical Implications

Given the significant relationship between medical consultation and better DM management, it would be beneficial to further educate diabetic patients regarding safe travel. This study suggests that safe travel practices can be reinforced through specific guidelines designed for such patients.

Educating patients on ways to monitor their blood glucose levels, adjusting their medication doses, preparing for emergencies when abroad, and the importance of carrying a medical ID is crucial. Moreover, providing a list of healthcare centers approved by the health ministry and Saudi embassy in every country, as well as information on where to get medications and receive medical care during emergencies, is just as important.

## Figures and Tables

**Table 1:** Sociodemographic and clinical characteristics of the included diabetic patients (*n* = 242).

Variable	*N* (%)
Age (mean ± SD)	51.58 ± 11.96
Duration of diabetes (mean ± SD)	12.75 ± 8.64
Gender (male)	65 (27.2)
Type of DM	
T1DM	37 (15.3)
T2DM	205 (84.7)
Occupation	
Unemployed	120 (50.8)
Ever employed	116 (49.2)
Income (SR)	
<5000	52 (21.5)
5000–<10000	58 (24)
10000–<15000	36 (14.9)
>15000	27 (11.2)
Don't know/don't want to answer	69 (28.5)
Smoking	
Nonsmokers	213 (88)
Ever smoked	29 (12)
Education	
School education	158 (65.3)
Higher education	84 (34.7)
Marital status	
Not married	76 (31.5)
Married	165 (68.5)
Medications	
Insulin users	105 (43.4)
Noninsulin users	137 (56.6)
Comorbidities	
Cardio vascular (yes)	53 (22.8)
COPD (yes)	27 (11.6)
Hypertension (yes)	115 (49.6)
Nephropathy (yes)	7 (3)
Thyroid (yes)	39 (16.8)
Musculoskeletal (yes)	11 (4.7)
Others	23 (9.9)
None	57 (24.6)
Last trip time	
Past 6 months	127 (52.5)
Past 6 months–1 year	71 (29.3)
Past 1-2 year	31 (12.8)
Past >2 years	13 (5.4)
Destination	
Domestic travel	145 (59.9)
International travel	96 (40.1)
Duration of last trip	
<1 month	205 (84.7)
1–6 months	37 (15.3)
Transpiration method	
Airplane	147 (60.7)
Car	91 (37.6)
Train	1 (0.4)
Physician telling about the importance of pretravel consolation visit (yes)	56 (23.7)
Travel-associated fears (yes)	36 (14.9)
Doctor's advice can help with fears	29 (82.9)

^*∗*^standard deviation.

**Table 2:** Knowledge and practice of patients with diabetes (*n* = 242).

Variable	*N* (%)
Knowledge	
Source of information	
Nurse	33 (14.1)
Books	19 (8.1)
Internet	55 (23.5)
Television	13 (5.6)
Others	14 (6)
None	124 (53)
Reason for pretravel doctor visit	
Travel distance	60 (25.9)
Travel duration	74 (31.9)
Travel risks	50 (21.5)
Other	26 (15)
Telling partner about condition (yes)	205 (87.6)
Changing insulin dose	
Correct answers	27 (11.2)
Source of care abroad	
Hotel staff	29 (12.2)
Embassy	45 (18.9)
General practitioner	132 (55.5)
Others	22 (9.2)
Source of medications abroad	
Hotel staff	8 (3.4)
Embassy	15 (6.4)
Pharmacy	141 (59.7)
General practitioner	35 (14.8)
Old prescription	38 (16.1)
Others	4 (1.7)
Practice	
Seeking medical consultation before travel	81 (33.6)
Reasons for consultation	
Monitoring	44 (53.7)
Insulin dose	27 (32.9)
Medication dose	27 (32.9)
Letter for insulin	7 (8.5)
Vaccinations	1 (1.2)
Travel medication	8 (9.8)
Destination	7 (8.5)
Others	1 (1.2)
Management issues (yes)	54 (22.5)
Issues	
Blood tests	10 (20)
Dosage	5 (10)
Meals	34 (68)
Others	5 (10)
Carrying identification	19 (7.9)
Carrying snacks	163 (67.6)
Carrying medications	230 (95)
Carrying a first aid kit	102 (42.3)
DM influences destination	31 (12.8)
DM influences mode of transportation	44 (18.2)
Self-monitoring	
More than usual	54 (22.5)
Per usual	133 (55.4)
Less than usual	53 (22.1)
Self-control	
Good	38 (15.8)
Per usual	76 (31.7)
Poor	126 (52.5)
Complications (yes)	50 (20.7)
Types of complications	
Hyperglycemia	26 (52)
Hypoglycemia	27 (54)
Wound inflammation	5 (10)
Others	1 (2)
Symptoms of infection	
Vomit	15 (6.2)
Diarrhea	15 (6.2)
Fever	15 (6.2)
Cough	32 (13.2)
Rhinitis	14 (5.8)
Skin rash	8 (3.3)
Others	9 (3.7)
None	160 (66.1)

**Table 3:** Association of categorical study variables with doctors visit of diabetic patients.

Variable	Doctors consultation (%)		*χ* ^2^ value	*p*-value	OR	95% CI of OR
	Yes	No				
Education						
Higher education	38 (45.2)	46 (54.8)	7.814	0.005	2.190	1.258–3.814
School education	43 (27.4)	114 (72.6)				
Doctor visit regarding duration						
Yes	34 (45.9)	40 (54.1)	7.794	0.005	2.253	1.266–4.010
No	43 (27.4)	114 (72.6)				
Doctor visit regarding possible risks						
Yes	26 (52.0)	24 (48.0)	9.143	0.002	2.637	1.390–5.002
No	53 (29.1)	129 (70.9)				
Telling partner about condition						
Yes	76 (37.3)	128 (62.7)	4.484	0.034	2.850	1.044–7.782
No	5 (17.2)	24 (82.8)				
Possible causes of glucose monitoring complications (meals related)						
Yes	10 (29.4)	24 (70.6)	4.963	0.026	0.250	0.071–0.875
No	10 (62.5)	6 (37.5)				
Carrying identification						
Yes	13 (68.4)	6 (31.6)	11.093	0.001	4.875	1.778–13.367
No	68 (30.8)	153 (69.2)				
Carrying a first aid kit during travel						
Yes	46 (45.1)	56 (54.9)	10.217	0.001	2.417	1.399–4.177
No	35 (25.4)	103 (64.6)				

Table 3. Variables: duration of diabetes, gender, type of diabetes, smoking, destination, marital status, type of medication, occupation, self-monitoring and control, doctors visit regarding distance, patients who had complications regarding (glucose monitoring and insulin dosage), complication during travel (hyperglycemia, hypoglycemia and inflammation) carrying snacks and medications, influence of diabetes on choosing destination and mode of transport were all non-significant in regards to doctor's consultation. OR, odds ratio; 95% CI, 95% confidence interval.

**Table 4:** Results of multi-variate logistic regression analysis.

Variables	*B*	*p*-value	OR	95% CI for OR
Concerns about duration	0.951	0.01	2.588	1.261–5.308
Concerns about possible risks	1.260	0.003	3.525	1.525–8.152
Carrying identification	2.221	0.003	9.214	2.137–39.732
Carrying first aid kit	0.717	0.027	2.048	1.085–3.867

OR, odds ratio; 95% CI, 95% confidence interval.

## Data Availability

The data used to support the findings of this study are available from the corresponding author upon request.

## Supplementary Materials

Supplementary Materials STROBE_checklist copy.
